# Hospital and Institutional News

**Published:** 1917-02-10

**Authors:** 


### HOSPITAL AND INSTITUTIONAL NEWS.

### THE GREAT CENTRAL HOTEL AS A CLEARING HOUSE FOR WOUNDED OFFICERS.

IT was announced some time ago in THE HOSPITAL that the Army Medical Department was taking over the Great Central Hotel to convert it into a convalescent hospital for wounded officers. The building has now been opened for this purpose, and it is understood that the name of the Prince of Wales is to be associated with it. Coincident with the provision of this hospital a change is taking place in the system of disposal of wounded officers in London. Officers are no longer to be allowed so many weeks or months of sick leave by a Medical Board, and permitted (once they are discharged from hospital) to spend the time at home or anywhere else they please. On discharge from hospital they will be sent to the Great Central Hotel, where they will be detained—in most cases for quite a short time, doubtless—until the authorities there decide whether they are fit for duty, light duty, convalescent hospital, or what not. The new system will result in a very considerable curtailment of the liberty which convalescent officers have hitherto enjoyed, and will not be at all popular in the Service. It is supposed that the reason for this drawing tighter the bonds of discipline is that a small proportion of officers given sick leave have abused their freedom, and have in certain cases actually damaged their health and thus delayed their return to useful employment by their conduct when at large. It is hard on the great majority, who have not converted liberty into licence; but the innocent have to suffer for the guilty, and there is no doubt that the new system will make for greater efficiency in the Army, and that is all that matters at the present time.

### LORD RHONDDA AND INFANT WELFARE.

A DEPUTATION from the Association of Infant Welfare and Maternity Centres, which represents the majority of the 800 existing Centres of this kind, was received by Lord Rhondda on Wednesday, January 31. The Right Hon. Arthur Acland, a member of the committee, suggested, in addition to the particular pressing reform which the deputation had come to urge, various future forms of State help which would materially strengthen the movement for saving the babies for the nation. The deputation particularly urged the extension of the present Government grant of 50 per cent. of the approved expenditure incurred by the Welfare Centres to cover the cost of supplying milk for children under school age and nourishment for expectant and nursing mothers in necessitous cases. Dr. Pritchard pointed out the difficulty, in existing circumstances, of providing the necessary 1½ pint of milk per day for babies that had to be bottle fed, and the still greater difficulty in affording as much milk as was really needed for children from nine months to three years, if they were to grow up sound and strong. He also stated that a small quantity of sugar, now difficult to get, was vitally necessary for children under eighteen months of age. Dr. Shepherd referred to the importance of providing nursing and expectant mothers with adequate nourishment, and stated that her experience of the provision of suitable meals at Centres for Mothers (supplied at a cheap rate where prescribed by the doctor) was that it increased largely the number of cases in which the babies were breast fed. In the course of a sympathetic reply, Lord Rhondda foreshadowed early legislation, as a war measure, with a view to increasing the powers of local authorities in helping the welfare of infants and of expectant and nursing mothers. He referred to the recommendations of the Food Prices Committee in favour of the supply of milk for infants and food for mothers as suggested by the deputation, and he hoped that the measures he was proposing would, with the active co-operation of local authorities and voluntary organisations, result in a very material saving of infant life.

### A MANY-SIDED CAREER.

A VERSATILE and widely-known retired officer of the Indian Medical Service passed away on February 2 in the person of Colonel Thomas Holbein Hendley, C.I.E., after a long illness, and in his seventieth year. Of recent years, since his retirement from the Service, Colonel Hendley has been conspicuous chiefly for his energies and enthusiasm in the work of the Ambulance Association of the Order of St. John of Jerusalem, of which Order he was a Knight of Grace; and in connection with the West London Medico-Chirurgical Society, of which he was a past president. In earlier days he was equally keen on the Volunteer movement. His chief claim to distinction, however, rests upon his studies of Indian art, in support of which he was unfailing and indefatigable. He organised first the Jaipur Exhibition and then the Jaipur Museum; wrote several volumes on various branches and aspects of native art; and was one of the originators of the *Quarterly Journal of Indian Art*, to which he was a voluminous contributor. Colonel Hendley received his professional education at St. Bartholomew's Hospital, where he was a Gold Medallist. He entered the Indian Medical Service in 1869, and retired in 1903. Twenty-four years of his career there were spent in Rajputana: after that he became for six years Inspector-General of Civil Hospitals in Bengal. Colonel Hendley had a wide knowledge of institutional affairs and management, not only in India, but in England also.

### A HOSPITAL SUPERINTENDENT RECEIVES THE C.B.

COLONEL D. J. MACKINTOSH, M.V.O., LL.D., whose restoration to health is matter for congratulation, is the able medical superintendent of Glasgow Western Infirmary. He has just been made a Companion of the Bath. Prior to his appointment to the Western Infirmary in 1892, he was in successive years resident surgeon of the Glasgow Eye Infirmary, senior resident medical officer at the City of Glasgow Fever Hospital, and medical superintendent of the Victoria Infirmary, Glasgow, so that his institutional experience has been considerable. He has devoted much attention to the construction and equipment of modern hospitals, and his book on this subject has reached a second edition. Colonel Mackintosh has always been keenly interested in the Territorials and ambulance work. From 1912 to 1916 he was Assistant-Director of Medical Services, Lowland Divisional area of Scotland, and from November 1914 till August 1916 he supervised the administration and organisation of all military, war, and Territorial general hospitals within the Glasgow area. He is on the Council or a committee member of various medical and nursing bodies, including the Royal British College of Nursing and its Scottish Board.

### A LONG CAREER OF INSTITUTIONAL SERVICE.

The death, at the age of eighty-one, of Mrs. John Walter, widow of the third of that name who was for nearly fifty years the chief proprietor of the *Times*, removes not merely one who was closely associated with Delane and other notable members of the staff of that great newspaper, but also was for many years a keen worker and committee-man in the London institutional world. Mrs. Walter joined the committee of the Florence Nightingale Hospital for Invalid Gentlewomen in 1873; and after her husband's death in 1894 she devoted even more of her time and labour towards promoting its interests than before. She was president of the committee for thirteen years, from 1893 to 1906; and had much to do with the successful removal of the hospital from its old home at 90 Harley Street to its present quarters at Lisson Grove. This hospital, as most of our readers are well aware, was founded by Lady Canning and Lady Waterford in 1850, with Miss Florence Nightingale as Lady Superintendent.

### A CHARGE OF FALSE PRETENCES.

In the United States they have coined a special word to indicate the practice of giving or taking illicit commissions on medical fees, or of dividing between two doctors a fee which the patient imagines is paid to one alone. This practice is named "dichotomy," and is justly reprobated by all high-minded medical men in America, as it is in this country. At various times medical associations and societies in the United States have discussed the prevalence of this brand of dishonesty, and have adopted measures designed to diminish or uproot it. In this country such suggestions are seldom heard: probably, let us hope, because the thing itself is much less common. That it does occur here and there it is impossible to deny, but that it prevails to any extent we do not believe. A logical extension of the principle involved has recently been the subject of a criminal charge in one of the Courts. As the matter has been referred to a higher tribunal it is impossible to deal with the circumstances and merits of the particular case. The allegations of the prosecution include a charge that a practitioner (not a registered one, be it noticed) told a patient that a certain surgeon's charge for an operation would be twenty-five guineas; and then paid the surgeon fifteen guineas only, retaining the balance. Many years ago a case of this same sort occurred within our knowledge, though it never came before a Court of Justice; but it is doubtless an even rarer sort of ill-doing than plain, ordinary "dichotomy." If this particular allegation is substantiated, it would be of interest to hear who the surgeon is and what he has to say on the subject of his commerce with an unqualified practitioner.

### CO-OPERATORS AND THE ROYAL LANCASTER INFIRMARY.

At the Royal Lancaster Infirmary on Saturday week, Mr. Gregory, J.P., president of the Co-operative Congress (which held its meeting in Lancaster last Whitsuntide), and other prominent co-operators attended at the Royal Lancaster Infirmary to present a piano for the use of the wards, as a memento of the Congress being held in Lancaster and as a memorial of the late Mr. Blandford, an ardent and conscientious co-operator. It was explained that the delegates at each Congress made a collection, which was devoted to leaving behind in the town a suitable memento. On this occasion a piano had been purchased for the use of the wards at the infirmary, and a cheque for £10 was handed over to the Royal Albert Institution for imbeciles of the seven northern counties. The president of the infirmary, in accepting the gift, said it would be very much appreciated. A piano for the use of the wards had been very much desired, but the committee had never felt justified in spending public money on such a luxury. The gift was therefore very timely. It was mentioned that the committee intended to procure a low trolley for the piano, so that it could be moved about easily from ward to ward, and the co-operators present at once undertook to pay for it, so that the gift might be theirs complete.

### THE YORK WORKPEOPLE'S FUND.

With natural elation the York Workpeople's Hospital Fund announces that it raised in the past year £2,325 for the York County Hospital. This is a record, but it would be necessary to compare the number of workpeople who have left the city with the number of munition and other workers whom the war has brought there to arrive at a proper estimate of the permanent progress made. We emphasise this point because in the past there has been slackness at York in organising the hospital's income, and we want what we may call the year's progress recorded to be maintained in the future when the changes occasioned by the war have ceased to operate. Meantime the County Hospital may take courage and see that the line of organisation is also the line of permanent financial success.

### IS AN AUDITOR'S REPORT CONFIDENTIAL?

The discontent with the Welsh National Memorial Association, to which previous reference has been made in The Hospital, is again evident. The Glamorgan Insurance Committee has decided to ask the Association for the reports of its auditors during the past three years. The contention of the Insurance Committee is that, as a contributory body, it is entitled to see not only how the money has been spent, but what remarks the auditors have made upon the disbursements. Since there is alleged to have been some delay in complying with the Insurance Committee's request, perhaps the Association takes the view that the auditors' reports are confidential? Such a view could be paralleled in the business of joint-stock companies, which often allow only a summary balance-sheet, giving totals but not details, to their shareholders. Does the same rule apply to institutional balance-sheets and auditors' reports?

### A COTTAGE HOSPITAL AND A SACRED WELL.

Some anxiety has been caused to the trustees of the Holywell Cottage Hospital through the misfortune fallen on the town by the disappearance of the water of St. Winifride's Well. The town's chief attraction for visitors has gone, and the committee fears that the income will suffer accordingly. We believe, however, that these fears in the long run will prove exaggerated, since hospital finance depends not so much on the casual visitor even to a sacred well, as upon the widow's cruse of good will and charity: a force as miraculous as the cruse in the Bible story, but as superior to chance and change as was the famous cruse itself. That the town has lost a source of income is an added reason why the Holywell Cottage Hospital must not be allowed to suffer.

### CHILDREN'S HOSPITALS: A PARTNERSHIP SCHEME.

We have often had occasion to comment on the good work performed by the Royal Country Hospital for Children, Heswall, Cheshire, and now a proposal is on foot which deserves to be carefully considered before it is derided as impracticable. We refer to the plan, put forward by the Country Hospital, for entering into partnership with the Liverpool Children's Infirmary, whereby the proposed establishment of an auxiliary country institution in connection with the infirmary might be obviated. To this end a formal appeal, which was to have been issued on behalf of the infirmary six months ago, was held back. Negotiations for hospital partnership or amalgamation are usually protracted and not always successful, and such has been the case in connection with the present scheme. Moreover, the infirmary's not unnatural desire for full independence has been encouraged by the offer of Miss M. C. Twigge and Miss Camenish to establish at their own expense a small country hospital at Thingwall Hall, near Barnston, Cheshire. These ladies are willing to work with the infirmary, but we hope they will be public-spirited enough to see, before deciding to do so, whether their aid cannot be used to further the partnership of the infirmary with the Country Hospital. Probably they, too, would prefer to "paddle their own canoe." But the problem is a public one, and when it is remembered that the infirmary has a deficit of over £2,000 on its ordinary revenue, it is clear that a partnership would probably prove more economical than the foundation of a third institution would be. We therefore hope that a conference may be arranged between the three parties to decide what is best, not for both, but for the children and for economical hospital administration.

### DISTRACTION FOR THE WOUNDED.

A lady's generous and thoughtful action in the interests of the wounded soldier patients was referred to at a meeting of the Cardiff Mental Hospital Committee. Lieut.-Colonel Goodall reported that of the 1,200 beds available at the Welsh Metropolitan War Hospital 980 were occupied; 300 are set apart for orthopædic cases, and the remainder for general surgery and medical cases. The excellent services of the teacher in fancy mat-making, basket-making, crewel-work, etc., was gratefully acknowledged, for the men had been occupied in their minds and kept pleasantly engaged in the wards. For this boon, it was stated, the committee, staff, and patients were indebted to the kindness of Miss England, The Parc, Llanishen, who paid the entire cost herself, and to whom a cordial vote of thanks was accorded. The committee were greatly interested in the patients' work in this direction, and later inspected examples which showed what proficiency some had attained.

### BURNING FATALITIES AT WEST BROMWICH.

The senior surgeon to the West Bromwich Hospital, Mr. T. Sansome, has been very outspoken at the local Coroner's Court on the subject of accidental burnings of children in that district. He has said that since October last thirty-three cases of burns in children have been admitted to the hospital, of which fourteen have been fatal: in addition, fifteen or sixteen cases of burning have been treated, but not admitted as in-patients. He has mad inquiries with a view to finding whether this extraordinary epidemic can be attributed to mothers having to work away from home, and learns that this is not the case. The accidents have been mainly due to carelessness and negligence of almost criminal dimensions. The Coroner was evidently impressed by this outspoken evidence, for he stated that he should report the whole question to the proper authorities. In one of the two inquests upon fatalities due to burns, held on January 30, he told the mother responsible for the child's accident that he did not believe her evidence; it was shown that her home was in a dirty and neglected condition, and she was severely censured. We cannot believe that this regrettable record of negligence and death is in any way due to premeditation, but it is obvious that many West Bromwich mothers are taking their responsibilities a great deal too lightly.

### FEES FOR HOSPITAL AMBULANCES.

The gratitude felt by patients when the governors of the Dumfries and Royal Galloway Infirmary provided a motor ambulance to convey patients to the hospital was chilled, in some cases, when a fee was charged for its use. Even so, we learn, the car is hardly paying its way, and no money is being put aside for depreciation. In the case of a public hospital, one for infectious diseases for example, which is kept up out of the rates, it is natural that no charge for the hospital ambulance should be made. But when a voluntary hospital provides a motor ambulance for the comfort of its patients, they, knowing how its income is derived, should not resent a reasonable charge. The only alternative, and perhaps a better one, would be to create a special ambulance fund, to which donations or subscriptions could be earmarked. We believe that the money would be subscribed if the cost of the ambulance could not be raised, or raised only under protest, by charging a fee to patients for its use.

### PARENTHOOD AND THE POOR-LAW VETO.

MR. T. PERCIVAL, president of the National Poor-Law Officers' Association, made a capital speech at the recent conference of Poor-Law officers at Sheffield in dealing with their two major grievances—insecurity of tenure and the ban on children to which married officers are subject. The second is a national problem now, and the present position a scandal. Mr. Percival pointed out that while the child-welfare movement is everywhere nominally supported, yet in the rate-supported public services hundreds of young people were debarred from marriage, and those who were married were forbidden to have children. The evil was accepted as a matter of course. Guardians did not realise the hardship—indeed, we may add, the cruelty—of this system, in which, as Mr. Percival tersely put it, "one child is considered a serious inconvenience; two an almost insurmountable obstacle; and three a disaster." The proper remedy is for the President of the Local Government Board to insist on a file of all Poor-Law advertisements being kept in his office, and then to issue a circular-letter dropping heavily on any Board of Guardians which limits its appointments, in the revolting phrase of Bumble, to those with "no encumbrances." When this has been done it will be time to raise the other conditions of Poor-Law appointments which make it virtually impossible for officers to marry and to have children.

### THE PLYMOUTH ROYAL EYE INFIRMARY.

LAST year the Plymouth Royal Eye Infirmary had a balance of income over expenditure of £126. This year there is a balance on the wrong side of the ledger of £40. The debit is not a very serious matter, and the Mayor, when commenting on it at the annual meeting, remarked that it had been found by experience that a balance at the bank was not a good thing for a charitable institution. Be that as it may, the value of the work done by the Plymouth Royal Eye Infirmary will surely always ensure for the institution an income sufficient to enable it to "carry on" with its accustomed efficiency. Those who know what it is accomplishing week in and week out through the whole year will not be disposed to quarrel with the decision arrived at by the committee, when the Red Cross Society asked that the infirmary might be handed over to them as a hospital. The committee replied that a cessation of the Royal Infirmary's work would be a calamity for the entire neighbourhood served by the institution—which includes Cornwall as well as Devon. The fact that the new patients in the year just closed totalled 2,250 indicates the important place which the Eye Infirmary fills in the West Countree.

### SECTARIAN BICKERING AT THE METROPOLITAN ASYLUMS BOARD.

SOME feeling was manifested at the meeting on February 3 of the Metropolitan Asylums Board over a proposal to allow children of any particular denomination to be removed from the Board's hospitals whenever a special certified institution has been provided by a religious body of the persuasion to which the children are affiliated. The proposal seems to have been introduced at the desire of the Roman Catholic authorities, and was objected to by one member of the Board on the ground that the Asylums Board would lose all the control over children suffering from certain diseases delegated to it by the Government. A Roman Catholic priest, who replied to this contention, imparted a good deal of warmth into his speech; and a subsequent speaker very rightly pointed out that the question is essentially a medical one, and deprecated the importation of sectarian jealousies and bickerings. In the event, the recommendation allowing the transfer of children in the circumstances indicated was duly approved by the Board.

### THIS WEEK'S DRUG MARKET.

THE main event of the week has been a material drop in the value of iodine, and consequently in the prices of potassium iodide, sodium iodide, and iodoform. Generally speaking, the market has been rather more active. An interesting question that is being discussed relates to the possible effect of the interruption of the Dutch traffic on the price of quinine; the price tendency at the moment is in an upward direction, but there is no anxiety to make purchases, which suggests that buyers are not alarmed. The scarcity of cocaine has become even more acute, and still higher prices are asked for any small parcels that are offered. The downward movement in the price of phenacetin continues. Salicylates are unchanged, and there is a feeling that any further reductions that may take place will be small. Acetylsalicylic acid is unchanged. Phenazone is again rather dearer. Resorcin continues very scarce. Sulphonal is considerably dearer, as only small supplies are available. Salol is rather dearer. Still higher prices are being paid for any small quantities of barbitone that are offered. Lithia citrate has been reduced in price. Makers of salicin have raised their quotations. The question of the supply of glycerine has reached an acute stage, and it is of the utmost importance that practitioners should prescribe this substance as sparingly as possible; no medicinal glycerine is to be manufactured, and such small quantities as may be allotted from existing stocks must be used economically; hospital pharmacists should preserve any supply they may have as carefully as if it were some precious substance; they should bring suitable substitutes to the notice of the medical staffs.

